# Dapsone-induced Methemoglobinemia in a Child with End-stage Renal Disease: A Brief Review

**DOI:** 10.7759/cureus.2513

**Published:** 2018-04-20

**Authors:** Madhuradhar Chegondi, Irina Ten, Balagangadhar Totapally

**Affiliations:** 1 Division of Pediatrics, University of Central Florida College of Medicine, Orlando, USA; 2 Department of Pediatrics, Herbert Wertheim College of Medicine Florida International University, Miami, USA

**Keywords:** dapsone, methemogobinemia, child

## Abstract

We report a child who was on immunosuppressive therapy for renal transplant rejection, with end-stage renal disease, and who developed methemoglobinemia while receiving dapsone for Pneumocystis jiroveci infection prophylaxis.

## Introduction

Methemoglobinemia (MHb) is one of the forms of dyshemoglobinemia and occurs due to congenital and acquired causes [1-2]. Oxidizing agents, such as dapsone, cause oxidation within the heme moiety of the hemoglobin molecule and result in MHb. The oxygen-carrying capacity of red blood cells (RBCs) is impaired due to MHb, and children with MHb clinically present with cyanosis [2].

## Case presentation

A four-year-old girl with a history of asphyxiating thoracic dysplasia (Jeune syndrome) and end-stage renal disease, with a post-renal transplant, was on immunosuppressive therapy for chronic rejection. She was admitted to our tertiary care pediatric intensive care unit (PICU) with cardiorespiratory failure. The child was intubated and started on mechanical ventilation. The child was on daily peritoneal dialysis for renal failure and on dapsone for Pneumocystis prophylaxis for the last six months. During the hospital stay, she developed multiple complications, including sepsis, electrolyte imbalance, and thrombocytopenia. She was on various medications, including fentanyl, midazolam, mycophenolate, prednisolone, pantoprazole, cefepime, and dapsone. During the third week of hospital stay, the child had diarrhea and then developed metabolic acidosis. She was also noted to have cyanosis, low oxygen saturation (82% to 87%) by pulse oximetry (peripheral capillary oxygen saturation (SPO2)), despite the escalation of inspired oxygen to 100%. A blood gas analysis consistent with metabolic acidosis (ph 7.23, oxygen partial pressure (PaO2) 125 mmHg, partial pressure of carbon dioxide in arterial blood (PaCo2) 42 mmHg, HCo3 14.6, bases excess (BE) -9). With cyanosis, low oxygen saturation by pulse oximetry (SPO2) in the setting of normal oxygen partial pressure (PaO2) methemoglobinemia was suspected. Co-oximetry was ordered, which revealed a methemoglobin level of 21.5% (normal range 0 to 2%). Laboratory workup ruled out hemolysis. Since the child had end-stage renal disease, methylene blue was not considered as a treatment option, and she was started on daily ascorbic acid 500 mg via a gastrostomy tube. Dapsone was discontinued and pentamidine nebulization monthly started for Pneumocystis prophylaxis. Serial methemoglobin levels on co-oximetry showed an improving trend. Within 48 hours, the methemoglobinemia resolved and the level came down to 0.4 mg/dl. Ascorbic acid was continued for four more days and discontinued. Off ascorbic acid, the methemoglobin level remained less than 0.3 mg/dl. After a meticulous review of the medications and workup, we concluded that dapsone was the probable cause of methemoglobinemia in this child.

## Discussion

Methemoglobin (MHb) is abnormal hemoglobin in which the ferrous form of hemoglobin is oxidized to the ferric form. Enzymatic systems at the cellular level usually undergo spontaneous oxidation, which is the process of auto-oxidation; this oxidation rate increases with endogenous or exogenous oxidant exposure [1]. Normally 1% to 2% hemoglobin is oxidized to form methemoglobin daily [2]. RBCs have developed multiple reducing systems to maintain a normal methemoglobin level. The most important mechanism is the reduced nicotinamide adenine dinucleotide-dependent cytochrome-b5-methemoglobin (NADH cytochrome-b5-MHb) reductase system [2]. Individuals with congenital methemoglobinemia are homozygous for NADH cytochrome-b5-MHb reductase deficiency, and they usually have a 10%-50% methemoglobin level even in the absence of oxidant stress [2]. Young infants are more susceptible to develop methemoglobinemia due to a lack of full activity of NADH cytochrome-b5-MHb reductase [2]. The other minor pathways involved in the reduction of methemoglobin are nicotinamide adenine dinucleotide phosphate methemoglobin reductase (NADPH-MHb reductase), glutathione, and ascorbic acid [2]. Hemoglobin M disease is another cause of congenital methemoglobinemia, which results from a single globin gene mutation. Reduced hemoglobin normally releases an oxygen molecule; occasionally, with autooxidation, an electron is also lost. This oxidation rate increases with hemoglobin M and other oxidant xenobiotics [2].

Most exogenous agents that are responsible for acquired methemoglobinemia alter the balance between hemoglobin and methemoglobin through their metabolites [2]. Drugs such as sulfonamides, nitric oxide, nitroprusside, chloroquine, primaquine, lidocaine, and prilocaine can result in methemoglobinemia [2]. Dapsone-induced methemoglobinemia may be dose-related or an idiosyncratic reaction in susceptible individuals [3]. Dapsone is well absorbed from the gastrointestinal tract, undergoes enterohepatic circulation, and is excreted through the kidneys. It has a long half-life of 24-30 hours, Dapsone metabolizes through acetylation and N-hydroxylation. Its metabolite dapsone hydroxylamine is a potent oxidant, responsible for adverse effects, including methemoglobinemia and hemolytic anemia [4]. In patients with renal failure, the impaired excretion of dapsone metabolites makes them more susceptible to methemoglobinemia [4]. The heterozygosity of NADH cytochrome-b5-MHb reductase may predispose to methemoglobinemia even with lower doses of dapsone [4]. Metabolic acidosis can produce methemoglobinemia by decreasing methemoglobin reductase [5]. Diarrhea, by altering the intestinal flora, increases the formation of nitrites from dietary nitrates. Nitrite reduction to ammonia is also impaired in diarrhea, which makes more nitrite available for intestinal absorption and the subsequent oxidation of hemoglobin to methemoglobin [5]. Although our patient was on dapsone for six months with no adverse effects, he developed methemoglobinemia due to concurrent diarrhea and metabolic acidosis. 

Symptoms of methemoglobinemia are related to impaired oxygen-carrying capacity and delivery to tissues. Cyanosis is a prominent finding in methemoglobinemia; it usually appears when the concentration is 15%-20%. Patients develop exertional dyspnea, headache, dizziness, and fatigue at the level of 20%-50%. Dysrhythmias, seizures, and coma can occur if the concentration is between 50% and 70%; above 70%, severe hypoxic symptoms and death can occur [2,6]. Dapsone-associated methemoglobinemia may also have concurrent hemolytic anemia [6]. In the presence of renal failure, drugs like dapsone can cause prolonged symptoms of methemoglobinemia [6].

 A clinician should suspect methemoglobinemia or other dyshemoglobinemias if there is a discrepancy between the PaO2 on blood gas analysis and oxygen saturation on pulse oximetry [2-3]. With methemoglobin levels above 30%, the pulse oximeter is very misleading, as SPO2 plateaus around 85% [1,7]. The presence of a saturation gap of 5 or more, which is a disparity between the measured oxyhemoglobin saturation of the pulse oximeter and the calculated oxyhemoglobin saturation of the arterial blood gas is an essential clue for the diagnosis of methemoglobinemia and other dyshemoglobinemias [3,7]. The definitive diagnostic test for methemoglobinemia is co-oximetry [7]. In contrast to pulse oximetry, co-oximetry measures methemoglobin, and carboxyhemoglobin, in addition to oxyhemoglobin and deoxyhemoglobin [7].

The treatment of methemoglobinemia includes the removal of any oxidative stress, supportive care, and the administration of methylene blue. In cases of mild methemoglobinemia, withdrawal of the offending agent is the only indicated treatment modality. In patients with abnormal vital signs or lactic acidosis due to tissue hypoxia, methylene blue is indicated. In the absence of clinical features, a methemoglobin level above 25% may warrant empiric therapy; however, levels below 25% warrant close observation [8].

Typically, methemoglobinemia is treated by removing the offending agent, supportive care, and administering methylene blue. Methylene blue acts as a cofactor in the NADPH pathway and accelerates the enzymatic reduction of methemoglobin. Its usual dose is 1-2 mg/kg IV; the treatment can be repeated in one hour, depending on the methemoglobin concentration [2]. Symptomatic improvement should occur within 30 minutes. If there is no response with two doses of methylene blue, consider an alternate diagnosis, such as glucose-6-phosphate dehydrogenase (G6PD) deficiency, NADPH-methemoglobin reductase deficiency, hemoglobin M, or sulfhemoglobinemia [8]. Rebound methemoglobinemia can be seen in drug-induced etiology, especially with drugs having long half-lives like dapsone, which requires either continuous infusion of methylene blue [9] or the use of activated charcoal to interrupt enterohepatic circulation [8]. Charcoal hemoperfusion can be used if activated charcoal is not tolerated [10]. Ascorbic acid can be used in patients with G6PD deficiency, where methylene blue is contraindicated, but it reduces methemoglobin level at a slower rate [8]. Cimetidine, which inhibits the cytochrome P450-mediated pathway, can be used prophylactically in patients on dapsone, to reduce the formation of dapsone metabolites and to increase the tolerability of high-dose dapsone [6]. Persistent methemoglobinemia requires exchange transfusion or hyperbaric oxygen therapy [8].

## Conclusions

Though the presence of central cyanosis is most often due to cardiorespiratory causes, alternative causes, such as methemoglobinemia, should always be considered. Dapsone is a well-established cause of methemoglobinemia; its occurrence is more common, especially in impaired renal clearance and with associated aggravating factors like diarrhea and metabolic acidosis. Rapid recognition, aggressive treatment, and serial measurements of methemoglobin levels on co-oximetry to monitor the therapeutic response are key factors in a successful outcome for these patients.
